# Cardiogenic Versus Obstructive Shock as a Consequence of Road Traffic Accidents: A Case Report on Approach

**DOI:** 10.1155/cric/9592779

**Published:** 2026-08-17

**Authors:** Azri Salih Haji Sgery, Philip Fathil Essa, Dilbar Lhon Sultan, Ahmed Mohammed Sharif, Abdullah Ibrahim Mohammed

**Affiliations:** ^1^ Azadi Emergency Hospital, Duhok, Iraq; ^2^ Azadi Hospital, Duhok, Iraq

**Keywords:** Beck′s triad, cardiac tamponade, case report, E-FAST, hemopericardium, road traffic accident

## Abstract

**Background and Introduction:**

Cardiac tamponade is a medical/traumatic emergency characterized by the increased intrapericardial pressure from the accumulation of excessive fluid (either exudate, transudate, or blood) in the pericardium. Emergency sonography is a vital part of the rapid assessment of cases with traffic accidents and chest traumas, as pericardial injuries are among the hidden affected organs. Here, we present a case of successful resuscitation of cardiac tamponade.

**Case Presentation:**

An 18‐year‐old male presented as a case of a traffic accident within 30 min with hemodynamic instability from bradycardia, hypotension, decreased Glasgow Coma Scale, and distended jugular venous pressure. Bilateral air entry was present, with the aid of E‐FAST, tension pneumothorax was excluded, and cardiac tamponade was diagnosed. Immediately, needle pericardiocentesis was done under the supervision of a senior traumatologist, followed by rapid improvement. A central venous line was inserted in the pericardium for continuous drainage. A total of 810 mL of blood and serosanguineous fluid were drained. The patient was discharged home after 4 days of intensive care stay and 1 day in the ward.

**Discussion:**

Beck′s triad, along with bradycardia, indicates cardiac tamponade until proven otherwise. Any case presenting with features of hypotension, especially following trauma to the chest, should have tamponade excluded by using E‐FAST. Needle pericardiocentesis is a life‐saving procedure that can provide immediate relief through aspiration of the pressure.

## 1. Introduction

The heart, despite the variable exact position, tends to lie fairly horizontally with its apex directed toward the left side of the body [1], located in the mediastinum between the third and sixth costal cartilage, and is surrounded by a protective membrane named the pericardium [1, 2]. The latter comprises an outer fibrous layer and an inner double‐layered serous pericardium. Within the double‐layered serous pericardium lies the pericardial cavity, which normally contains 15–50 mL of ultrafiltrate plasma [2].Accumulation of the fluids in the pericardial cavity, which could be either transudate, exudate, or sanguineous, is known as pericardial effusion [3]. The disease could happen from an infection, inflammation, idiopathic causes, malignancy, or direct filling of the sac by blood from a defect in the myocardium, such as iatrogenic or traumatic cardiac wall injury, backflow from aortic dissection [3]. Once the fluid accumulation impacts the cardiac function, it becomes known as Cardiac tamponade, which is a medical or traumatic emergency characterized by the increased intrapericardial pressure from the accumulation of excessive fluid (either exudate, transudate, or blood) in the pericardium over a period of time [2, 4]. The disease poses a life‐threatening cardiac event on patients′ lives through restricting the filling of cardiac chambers, leading to hypotension, decreased cardiac output, and cardiac arrest [2].

Causes of tamponade can occur either in a slow pace of fluid effusion—such as infection in tuberculosis, autoimmune diseases, uremia from renal failure, and neoplasms—or at a more rapid pace—such as hemorrhage from penetrating wounds to the heart or rupture of ventricles as a consequence of myocardial infarction [5, 6].

Traumatic causes can cause hemodynamic instability with a smaller amount of fluid [5]. Patients typically present with features of cardiogenic or obstructive shock. Other features include chest pain, shortness of breath, dizziness, and syncope. More severe presentations include features of Kussmaul sign, Ewart′s sign, pulsus paradoxus, and change in mental status. Nonetheless, it can cause pulseless electrical activity cardiac arrest [5]. The classical physical finding of cardiac tamponade includes hypotension, distention of jugular venous pressure, and muffled heart sounds; these are known as Beck′s triad [5]. As its presentation is sudden and rapid, with the high fatality of the disease, diagnosis is usually made at postmortem [4]. However, emergency ultrasound is a vital part of rapid assessment of cases with traffic accidents and chest traumas [7], as pericardial injuries are among the hidden affected organs and will not be obvious on assessment.

Here, we present an 18‐year‐old male victim of a road traffic accident who presented with haemodynamic instability and was diagnosed with cardiac tamponade.

## 2. Case Presentation

**Figure 1:**
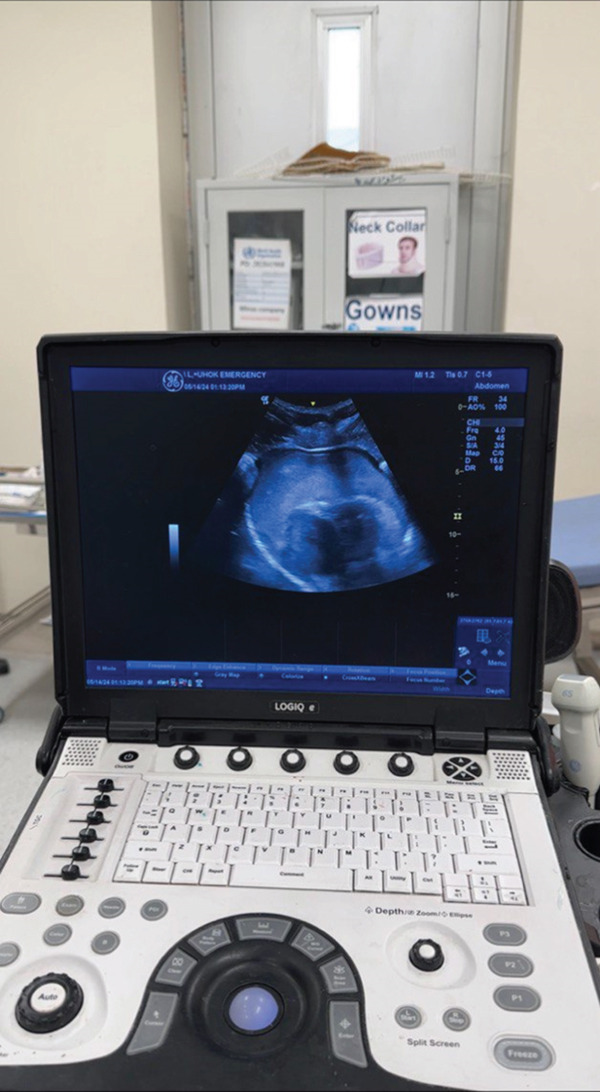
Cardiac tamponade illustrated on ECHO cardiogram initially at the diagnosis.

An 18‐year‐old male with a negative past medical and surgical history presented as a case of road traffic accident (RTA), ejected from a motorcycle after a collision with a car. On presentation to the trauma/resuscitation room, he was disoriented, and his level of consciousness was gradually deteriorating. He developed shortness of breath and bleeding from the mouth while he was on the trauma bed. His vitals initially were as follows: SpO_2_: 70% O_2_; pulse rate: 45 beats per min (bradycardia), blood pressure: undetectable, random blood sugar: 200 mg/dL, Glasgow Coma Scale was 7/15, pupils equal in size and bilaterally reacting to light. Advanced Trauma Life Support Protocol was initiated, and resuscitation was started. Upon further examination, he was found to have distended jugular venous pressure, with bilateral equal air entry on both sides. The diagnosis was focused on obstructive shocks, and cardiac tamponade had to be ruled out. Hence, an extended focused assessment with sonography in trauma E‐FAST was performed and showed massive cardiac tamponade Figure 1); otherwise, examinations were negative for collections in the abdomen or pneumothorax.

**Figure 2:**
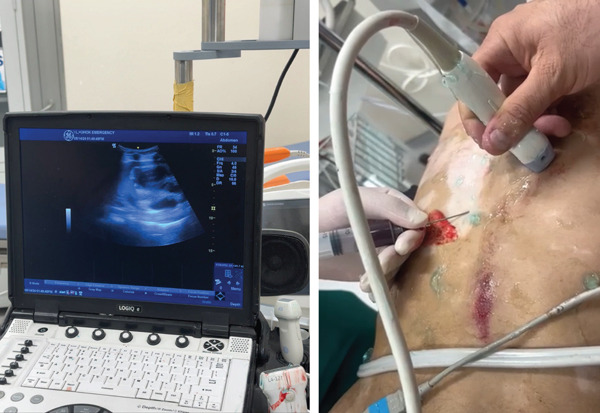
Following the pericardiocentesis.

**Figure 3:**
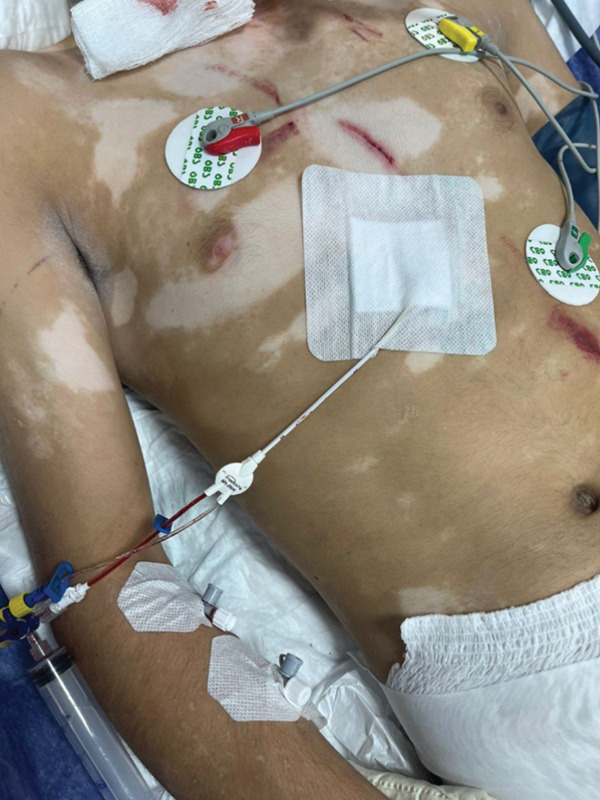
Central venous line inside the pericardial cavity.

Under the supervision of a senior traumatologist, an immediate needle pericardiocentesis under ultrasonographic guidance was done by a junior house officer. The process was preceded by decontamination of the field with iodine, and under aseptic technique, the central venous line (CV line) needle was inserted in the subxiphoid region directly toward the left shoulder at 30°. Under negative pressure, advancement of the needle was done until blood was seen and the needle was visible under the sonography. Aspiration of 20 mL of blood was done initially, and a rapid response was noticed; the heart rate went from bradycardia to tachycardia (40–130 s beats per min). Another 40 mL was aspirated and showed a significant improvement Figure 2. These steps were followed by the placement of a two‐way CV line inside the pericardium with a size 7 French and fixed at 10 cm for the continuous drainage Figure 3. A total of 200 mL of blood was aspirated within 10–20 min. Further resuscitation was done with 1 L of normal saline, followed by blood preparation and giving 2 pints of matching packed red blood cells as a total. The patient was intubated during the procedure. Antibiotics were covered with Meropenem 1 g three times daily, vancomycin 1 g twice daily, and flaggy bottle 500 mg three times daily. After resuscitation, the case was taken for computed tomography (CT) scans and was immediately transferred to the intensive care unit. Prior to reaching the ICU, a total estimation of ~280 cc of blood was aspirated. The heart contractility was re‐examined with the pericardial space and showed improvement. The patients were under close monitoring. He was extubated the day after with full recovery of consciousness and neurological functions. The CV line was removed after 3 days; Day 1 postplacement drained ~480 mL of blood. Day 2, the drainage became more serious and drained ~50 mL. On Day 3, the drain was removed. The patient was consulted for a mandibular fracture by the faciomaxillary surgeon and was to be operated on as soon as possible. During the ICU stay, his procalcitonin rose to 2.2, and with the same antibiotic coverage, it tapered down on the 2nd and 3rd day of hospitalization. A total of 810 mL of fluids were drained from that case. The patient remained in ICU for 4 days, 1 day in the ward, and was discharged home with a normal ECHO cardiogram and home treatment. He was prescribed Augmentin (1 g twice daily) and paracetamol tablets (1 g twice daily), with follow‐up after 1 week.

## 3. Discussion

Cardiac tamponade is a life‐threatening emergency characterized by the progressive accumulation of fluid or air in the pericardium, causing cardiac arrest within a short period of time through compressing the heart chambers [8]. Cardiac tamponade is a clinical diagnosis that requires a high index of suspicion for diagnosis via echocardiography/sonography and is managed with echocardiography‐guided pericardiocentesis [5, 8]. Immediate death can take place before even reaching the source of deterioration and can even lead to death [9], for which later identification would be useless for the patient. The diagnosis requires clinical evaluation: electrocardiography (ECG), focused assessment with sonography for trauma (FAST), chest x‐ray, echocardiography, CT, and cardiac magnetic resonance imaging (MRI) [10].

Cardiac tamponade due to trauma is more likely to occur following penetrating injuries than blunt traumas: about 80%–90% in penetrating injuries compared with 10% in blunt injuries [9], which could be one of the factors that can lead to missing the case and not diagnosing it. Most of the cases die at the time of accidents [9]; however, our case was lucky and presented to the hospital while in the stage of deterioration. During the assessment, he was entering bradycardia, and cardiac arrest was imminent, with emergency pericardiocentesis [10] and relief of the obstructive shock. Delayed cardiac tamponade can take place following chest trauma over 8 days to 4 weeks [9, 11].

The availability of bedside sonography has made it easier to detect collections within the pericardial cavity. The degree of pericardial effusions can be assessed on echocardiography by assessing the degree of thickening. Mild pericardial effusions will be seen posteriorly and mark < 10 mm in thickness, moderate effusions will be seen posteriorly and may be circumferential with a thickness of 10–20 mm, whereas large effusions mark > 20 mm in thickness and are circumferential [12]. In our case, the patient was in cardiac tamponade with the fluid covering the heart circumferentially. The case was classified as large effusions with a thickness of 25 mm.

Upon the patient′s assessment in the trauma bay, as information was collected, we noticed the patient to be in bradycardia and hypotension, that is, shock, in addition to a distended JVP. This information, along with the history of RTA, guided the diagnosis to the second fastest type of shock, which is obstructive shock; there will be decreased venous return to the right side of the heart due to external compressions [13]. Pneumothorax was ruled out due to bilateral equal air entry, and cardiac tamponade became most likely with two of the three criteria of Beck′s triad [5]. During E‐FAST, the diagnosis was confirmed.

In a retrospective study conducted from 1999 to 2015, studying blunt traumatic cardiac rupture, 76.2% of the cases survived, and 24% died postoperatively. The most common cause reported was traffic accidents, 81%; tamponade was suspected in 47.6% of the cases [14]. The incidence of traumatic cardiac tamponade is very low, up to 2%. Nevertheless, the mortality is relatively high and can reach 28.5% [7].

Two factors affect the diastolic filling and the development of symptoms: the rate of fluid accumulation and the compliance of the pericardium [12].

Management of such a life‐threatening event starts with the resuscitation protocol, securing the airway and providing oxygen, followed by providing two large‐bore cannulas and volume resuscitation, and searching for the cause of shock. Once the cause has been identified, treatment starts [15].

Treatment of life‐threatening traumatic pericardial effusions requires an immediate intervention with opening the pericardial sac through needle pericardiocentesis and the aspiration of the blood [5]. The rapid accumulation of blood in the pericardial space had led to an increased intrapericardial pressure, which exceeds central venous pressure, resulting in decreased venous return to the heart [6]. Other supportive measures include oxygenation and securing the airway, volume expansion, preparation of blood, and rapid sequence induction for intubation [15].

The use of triple antibiotics in this case was to prevent/minimize any chance of infection and protect the patient. However, he still developed sepsis inside the intensive care unit. Nonetheless, continuing with the same antibiotics led to improvement.

Beck′s triad is known to consist of muffled heart sounds, hypotension, and distended JVP. In cases of shock, the heart rate rises; however, in this case, there was bradycardia. The latter was caused by the pressure over the heart, impairing the contractility and decreasing the heart rate. Thus, when the aspiration was done with an acceptable amount, the heart rate increased and became tachycardic thereafter.

In any case following trauma, a rapid assessment of the heart with FAST is essential for pericardial fluid collection. Once the patient develops hemodynamic instability, such as tachycardia and hypotension, it becomes vital to exclude pericardial effusion or tamponade as cases might undergo management without identifying the source of injury, leading to delayed diagnosis and increasing the mortality [16].

## 4. Proposal

We would like to propose a new sign for a straightforward diagnosis. Once a patient presents with hypotension and bradycardia with a history of trauma to the chest, examine for JVP and muffled heart sounds and perform an E‐FAST. Beck′s triad, along with bradycardia, indicates cardiac tamponade until proven otherwise.

## 5. Conclusion

Cardiac tamponade is a medical/traumatic emergency that is almost invariably fatal. It requires a high index of suspicion due to the high fatality of the condition, with rapid action to relieve the pressure either surgically or through needle pericardiocentesis. Time plays a crucial role for management; the longer the delay, the worse the outcome. The mortality from hidden, traumatic cardiac tamponade is very high. However, the rapid action by the team has led to a successful identification of the cause of shock, rapid management, and saving the life of the patient.

## Author Contributions

A.S.H.S. did the pericardiocentesis under the supervision of A.I.M. The resuscitation was done by A.S.H.S., P.F.E., and D.L.S. under the supervision of A.I.M. The case was later followed up closely by A.M.S. The first draft of the article was written by A.S.H.S.

## Funding

No funding was received for this manuscript.

## Disclosure

All authors contributed to the final review and agreed on publishing. The work has been reported in line with the SCARE criteria [17].

## Ethics Statement

Ethical approval was provided by author′s institution.

## Consent

Written informed consent was obtained from the patient/legal guardian for publication of this case report and accompanying images. A copy of written consent is available for review by the editor‐in‐chief of this journal on request.

## Conflicts of Interest

The authors declare no conflicts of interest.

## Data Availability

Data sharing is not applicable to this article, as no datasets were generated or analyzed during the current study.
